# Evaluation of adherence to hand washing among health professionals: spy project

**DOI:** 10.1186/1753-6561-5-S6-P102

**Published:** 2011-06-29

**Authors:** SS Lessa, PA Oliveira, PR Daher, CA Binelli, AVD Silva

**Affiliations:** 1 1Infection Control Department, Hospital São Camilo, São Paulo, Brazil

## Introduction / objectives

Healthcare-associated infections affect hundreds of thousands of patients worldwide every year. Hand washing is a basic measure to reduce infections. The aim of this study was to evaluate the adherence to hand washing opportunities among the multidisciplinary team and to search for possible causes or situations that might promote hospital infections, including cross infection and infection outbreaks, among others.

## Methods

The methodology used was a descriptive, quantitative method that took place in a midsized charity institution in the city of São Paulo, Brazil. For data collection a "check-list" containing 7 closed questions was created. From May to June and November to December, 2010, a professional from the Hospital Infection Control Service performed observational visits in inpatient units and intensive care units, covering the morning, afternoon and evening periods of the day. The professional observed if doctors, physiotherapists and nursing staff washed their hands before and after completing their activities.

## Results

From the total of 758 opportunities for hand washing, the correct procedure was completed 491 times (65%). Considering the categories of health workers, physiotherapists washed their hands in 72% of the opportunities; nursing staff in 73% of the opportunities; and doctors in 40% of opportunities.

## Conclusion

In conclusion, this project identified important issues such as opportunities to improve infection control. By observing the results, we found that hand washing is not performed at every opportunity, which can increase hospital infections. It is not enough for just one team to have a correct practice, but it is necessary to understand the importance of meeting the prevention of infection recommendations.

## Disclosure of interest

None declared.

